# Heterogeneity of cough hypersensitivity mediated by TRPV1 and TRPA1 in patients with chronic refractory cough

**DOI:** 10.1186/s12931-019-1077-z

**Published:** 2019-06-06

**Authors:** Li Long, Hongmei Yao, Jing Tian, Wei Luo, Xinxin Yu, Fang Yi, Qiaoli Chen, Jiaxing Xie, Nanshan Zhong, Kian Fan Chung, Kefang Lai

**Affiliations:** 1 State Key Laboratory of Respiratory Disease, National Clinical Research Center for Respiratory Disease, Guangzhou Institute of Respiratory Health, The First Affiliated Hospital of Guangzhou Medical University, 151 Yanjiang Rd, Guangzhou, 510120 People’s Republic of China; 2 National Heart and Lung Institute, Imperial College London, London, UK; 3 Royal Brompton and Harefield Foundation NHS Trust, London, UK

**Keywords:** TRPV1, TRPA1, Cough phenotypes, Chronic refractory cough, Cough hypersensitivity

## Abstract

**Background:**

The differential sensitivity of cough to antitussive therapies implies the existence of heterogeneity in cough hypersensitivity, but how such heterogeneity is expressed across individual patients is poorly understood. We investigated the phenotypes of cough hypersensitivity by examining transient receptor potential ankyrin 1 (TRPA1)- and transient receptor potential vanilloid 1 (TRPV1)-mediated cough sensitivity in patients with chronic refractory cough.

**Methods:**

Using a selective TRPA1 agonist, allyl isothiocyanate (AITC), we established an AITC cough challenge as a measure of TRPA1-mediated cough sensitivity. The AITC cough challenge and the widely used capsaicin (a selective TRPV1 agonist) cough challenge were performed with 250 patients with chronic refractory cough and 56 healthy subjects. The concentration of AITC or capsaicin solution causing at least two (C2) and five coughs (C5) was recorded. Cough sensitivity was expressed as the mean (95% confidence interval) of log C5, and cough hypersensitivity was defined as a log C5 value lower than that of healthy subjects.

**Results:**

A distinct concentration–response effect of inhaled AITC was identified both in patients with chronic refractory cough and in healthy subjects. Cough sensitivity to AITC and capsaicin was significantly higher in patients than in healthy subjects (AITC: 2.42 [2.37–2.48] vs 2.72 [2.66–2.78] mM, *p* = 0.001; capsaicin: 1.87 [1.75–1.98] vs 2.53 [2.36–2.70] μM, *p* = 0.001) and was higher in females than in males for both healthy subjects and patients (all *p* < 0.05). Among the 234 patients who completed both challenges, 25 (10.7%) exhibited hypersensitivity to both AITC and capsaicin, 44 (18.8%) showed hypersensitivity to AITC only, 28 (11.9%) showed hypersensitivity to capsaicin only, and 137 (58.6%) exhibited hypersensitivity to neither. Those with TRPA1- and/or TRPV1-mediated hypersensitivity were predominantly female, while those without TRPA1- and TRPV1-mediated hypersensitivity were mainly male.

**Conclusions:**

Four phenotypes of cough hypersensitivity were identified by the activation of TRPV1 and TRPA1 channels, which supports the existence of heterogeneity in cough pathways and provides a new direction for personalized management of chronic refractory cough.

**Trial registration:**

ClinicalTrials.gov NCT02591550.

## Background

Chronic cough is a frequent condition seen by general practitioners and respiratory specialists. The most common aetiologies of chronic cough are asthma, eosinophilic bronchitis, upper airway cough syndrome, and gastro-oesophageal reflux disease [1, 2]; in patients with these aetiologies, the available therapies often provide adequate relief. However, in 20–46% of patients presenting to specialist cough clinics, the cough persists despite following guideline-based management protocols [3]. Such patients who experience marked adverse quality of life [4] have been defined as having chronic refractory cough. Treatment options for chronic refractory cough remain limited, mostly due to a poor understanding of the pathophysiological processes underlying the condition. The differential sensitivity of cough to antitussive therapies implies that the heterogeneity of the mechanisms underlying chronic refractory cough could be a contributing factor [5], but how such heterogeneity is expressed across individual patients is poorly understood.

Irrespective of the causes, patients with chronic cough suffer from irritating bouts of coughing evoked by low-level thermal, mechanical or chemical exposure, suggesting an abnormal upregulation of the cough reflex. Cough hypersensitivity syndrome (CHS) has been proposed to describe this condition [6]. Since activation of peripheral sensory nerves is usually the initiating factor that drives cough, the afferent limb of the cough reflex may represent an attractive target for the development of antitussive agents. There is growing evidence of roles of transient receptor potential vanilloid 1 (TRPV1) and transient receptor potential ankyrin 1 (TRPA1) in peripheral sensitization [7].

Capsaicin, a selective TRPV1 agonist, is most widely used to evaluate cough reflex sensitivity. Patients with chronic cough show heightened sensitivity to inhaled capsaicin, which improves after successful treatment. In addition, an increased expression of TRPV1 has been found in the epithelial nerves of the bronchial submucosa of patients with chronic cough, and this increased expression was positively correlated with capsaicin cough sensitivity [8]. However, in previous studies, capsaicin cough hypersensitivity has been reported to be absent in 50% of chronic cough patients [9–11].

Inhalation of TRPA1 agonists such as allyl isothiocyanate (AITC) and cinnamaldehyde can elicit a robust and repeatable cough response in rodents and healthy volunteers [12, 13]. However, whether TRPA1-mediated cough hypersensitivity is present in patients with chronic cough remains unknown. In a preliminary study, we utilized cinnamaldehyde as a cough-inducing agent and found it to be poorly-tolerated by patients with chronic refractory cough. In addition, the concentration required to cause 5 or more coughs (C5) was achieved in only 60% of subjects using up to the maximal inhaled concentration (800 mM). In contrast, AITC caused fewer side effects, and most subjects (94%) reached C5 (unpublished data). AITC appears to be a more selective TRPA1 agonist, given that a TRPA1 antagonist only partially inhibited cinnamaldehyde-induced cough but completely abolished AITC-induced cough in guinea pigs [14]. We therefore used AITC to investigate TRPA1-mediated cough sensitivity and used capsaicin to investigate TRPV1-mediated cough sensitivity, thus allowing us to determine the hypersensitivity status of these two different transient receptor potential (TRP) channels in patients with chronic refractory cough.

## Methods

### Subject recruitment

We recruited patients with a cough duration longer than 8 weeks that was unexplained after systematic investigation and treatment based on best-practice guidelines in our specialist cough clinic [15, 16]. These patients had clinical features indicating a state of cough hypersensitivity, namely, uncontrollable bouts of coughing triggered by low-level physical or chemical stimuli [7]. The healthy controls did not report any cough nor had signs of cough hypersensitivity. All participants were current non-smokers and had normal chest radiography and spirometry. Subjects who had an upper respiratory tract infection within 4 weeks or who took antitussive medications within 2 weeks of the study were excluded.

### Study design

AITC and capsaicin cough challenge were performed in a random order separated by an interval of at least 3 days. Subjects were blinded to the challenging agents. Spirometry (COSMED-Quark PFT, Milan, Italy) was assessed before and after each challenge in 25 patients and 25 healthy subjects in a preliminary experiment.

A stock solution of AITC (1000 mM, Xiya Reagent, Chengdu, China) was diluted with 50% ethanol to generate progressively increasing concentrations: 31.3, 62.5, 125, 250, 375, 500, 750 and 1000 mM. A 50% ethanol solution was used as a control solution. Capsaicin cough challenge was performed with capsaicin (Sigma Aldrich, Seattle, USA) solutions beginning at a concentration of 1.95 μM and then doubling the concentration up to 1000 μM as described previously [15, 17].

Concentration–response challenges to inhaled AITC and capsaicin were undertaken with a compressed air-driven nebulizer controlled by a breath-activated dosimeter (output of 0.025 ml per inhalation). Subjects were instructed to exhale to functional residual capacity and then inhale through a mouthpiece for 1 s. Each subject inhaled increasing concentrations of AITC or capsaicin solution at 1 min intervals. Coughs were counted during the first 30 s after inhalation. The lowest concentrations of AITC or capsaicin that evoked two (C2) or five (C5) coughs were determined.

We divided patients with chronic cough into high and normal cough sensitivity groups depending on whether their C5 value was lower than that of the control subjects [9–11]. For AITC, the log C5 value of healthy subjects was > 2.1 mM, so hypersensitivity to AITC was defined when log C5 was ≤2.1 mM. For capsaicin, the log C5 value of the healthy subjects was > 0.89 μM, so hypersensitivity to capsaicin was defined as log C5 ≤ 0.89 μM.

### Statistical analysis

Statistical analysis was performed using SPSS 18.0 (SPSS Inc., Chicago, USA). The C2 and C5 values were logarithmically transformed (to base 10). A paired t-test was used to compare spirometry before and after the cough challenge test. An unpaired t-test was used for two-group comparisons. Correlations between log C2 and log C5 of AITC and capsaicin values were analysed using the Pearson product-moment correlation coefficient. A *p* value < 0.05 was considered statistically significant.

## Results

### Clinical characteristics of subjects

A total of 250 patients with chronic refractory cough and 56 healthy subjects were enrolled in the study. All patients and healthy subjects completed the AITC cough challenge. A total of 234 out of the 250 patients completed the capsaicin cough challenge since inhalation of normal saline induced two coughs or more in 11 patients, and 5 patients dropped out after they underwent the first challenge test. Fifty-three out of the 56 healthy subjects completed the capsaicin cough challenge since 3 healthy subjects dropped out after they underwent the first challenge test (Table 1).

**Table 1:** Clinical characteristics of subjects

	Patients with CRC	Healthy subjects
Total number	250	56
Female (%)	133 (53.2)	27 (48.2)
Age (years)^a^	42.2 ± 13.9	36.3 ± 12.6
Cough duration (months)^b^	24 (2–480)	0 (0–0)
Daytime cough score^a^	2.7 ± 0.9	0.0 ± 0.0
AITC challenges, n	250	56
Capsaicin challenges, n	234	53
Cough triggers, n (%)		
Odours	165 (66.0)	0 (0)
Cold air	122 (48.8)	0 (0)
Dusty environment	116 (46.4)	0 (0)
Talking	100 (40.0)	0 (0)
Laryngeal paresthesia, n (%)	190 (76.0)	0 (0)

^a^Data are expressed as mean ± SD; ^b^Data were expressed as median (range); *CRC* chronic refractory cough

### AITC and capsaicin cough challenge

**Fig. 1:**
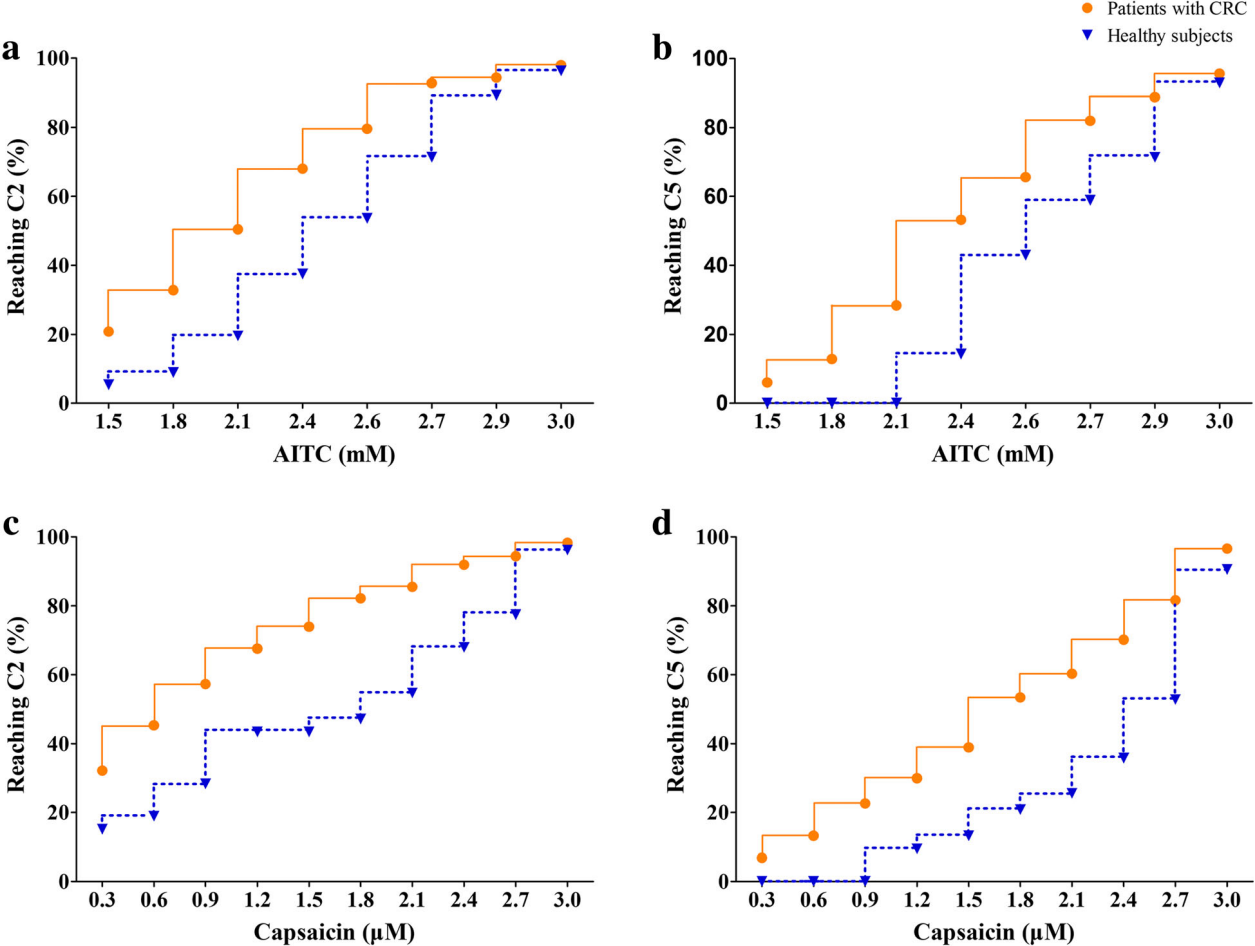
Concentration-dependent induction of coughs to reach either C2 or C5. AITC cough challenge (Panels **a** and **b**), capsaicin cough challenge (Panels **c** and **d**). C2: the lowest concentrations of AITC or capsaicin which evoked two to four coughs; C5: the lowest concentrations of AITC or capsaicin which evoked five coughs or more. The solid lines with dots represent patients with chronic refractory cough (CRC) and the dashed lines with triangles represent healthy subjects

During the AITC cough challenge, 245 (98.0%) patients and 54 (96.4%) healthy subjects reached C2, and 239 (95.6%) patients and 52 (92.9%) healthy subjects reached C5. During the capsaicin cough challenge, 230 (98.3%) patients and 51 (96.2%) healthy subjects reached C2, and 226 (96.6%) patients and 48 (90.6%) healthy subjects reached C5. The number of subjects who reached C2 and C5 increased with increasing inhaled concentrations of AITC and capsaicin (Fig. 1).

**Fig. 2:**
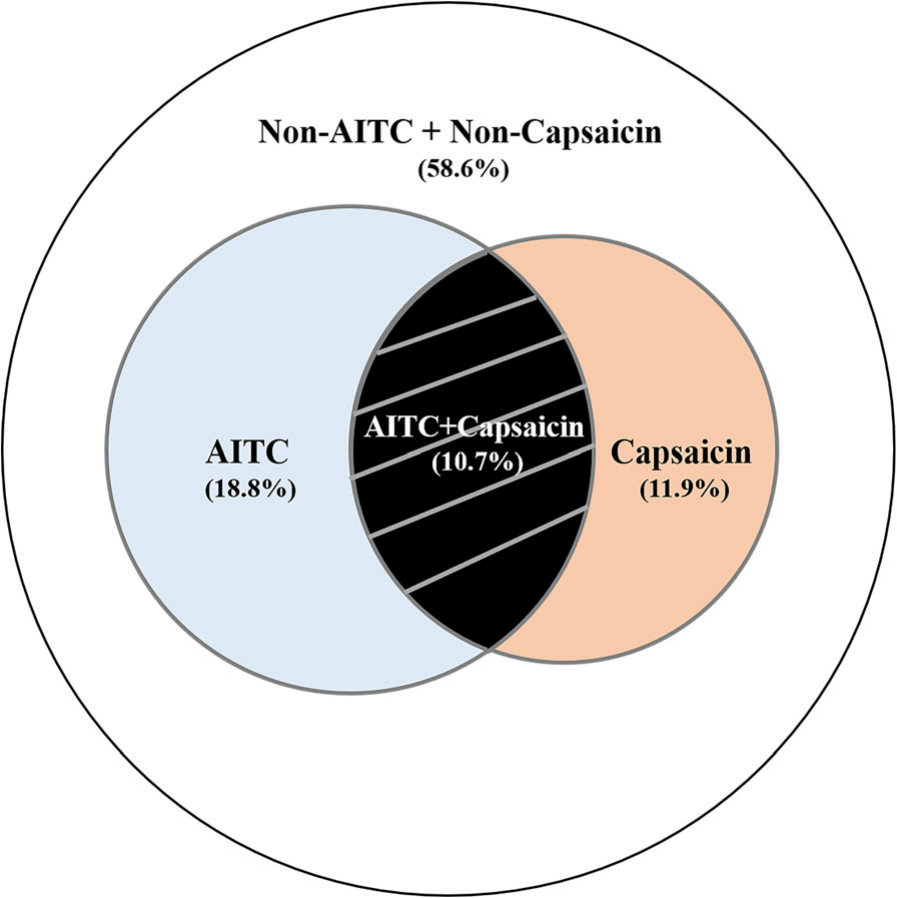
Venn diagram showing the group that are hypersensitive to TRPV1 (capsaicin), TRPA1 (AITC)

Among the 234 patients who finished both the AITC and capsaicin challenges, 25 (10.7%) had hypersensitivity to both AITC and capsaicin, 44 (18.8%) showed hypersensitivity solely to AITC, 28 (11.9%) demonstrated hypersensitivity solely to capsaicin, and 137 (58.6%) had no evidence of heightened sensitivity to either capsaicin or AITC (Table 2 and Fig. 2).

**Table 2:** TRPA1-, TRPV1-mediated cough hypersensitivity in patients with chronic refractory cough

		Capsaicin		Total
		+	–	
AITC	+	25 (10.7)	44 (18.8)	69 (29.5)
	–	28 (11.9)	137 (58.6)	165 (70.5)
Total		53 (22.6)	181 (77.4)	234 (100)

Data are expressed as n (%). +: Increased cough sensitivity to AITC or capsaicin; −: Normal cough sensitivity to AITC or capsaicin

Patients with heightened cough sensitivity to capsaicin, AITC or both were predominantly female, while patients with no evidence of heightened cough sensitivity to capsaicin or AITC were predominantly male. Patients with different types of cough hypersensitivity exhibited a similar proportion of sensitivity to different cough triggers and laryngeal paraesthesia (Table 3).

**Table 3:** Clinical characteristics of patients with different phenotypes of cough hypersensitivity

	AITC (+) Capsaicin (+)	AITC (+) Capsaicin (−)	AITC (−) Capsaicin (+)	AITC (−) Capsaicin (−)	*P*
Female^a^	19 (76.0)	25 (56.8)	20 (71.4)	56 (40.8)	0.001
Age (years)^b^	42.0 ± 11.5	44.0 ± 13.5	44.5 ± 13.1	41.3 ± 13.5	0.909
Cough duration (months)^c^	12.0 (2.0–480.0)	14.5 (2.0–360.0)	21.0 (2.0–480.0)	18.0 (2.0–480.0)	0.664
Daytime cough score^b^	2.5 ± 1.0	2.6 ± 0.7	2.9 ± 0.9	2.7 ± 0.8	0.371
Laryngeal paresthesia^a^	19 (76.0)	38 (86.4)	23 (82.1)	107 (78.1)	0.627
Cough triggers^a^					
Odours	19 (76.0)	27 (61.4)	23 (82.1)	92 (67.1)	0.237
Cold air	12 (48.0)	17 (38.6)	16 (57.1)	65 (47.4)	0.491
Dusty environment	11 (44.0)	19 (43.2)	16 (57.1)	58 (42.3)	0.551
Talking	10 (40.0)	23 (52.3)	12 (42.9)	45 (32.8)	0.131

^a^ Data are expressed as n (%); ^b^ Data are expressed as mean ± SD; ^c^ Data were expressed as median (range);

+: Increased cough sensitivity to AITC or capsaicin; −: Normal cough sensitivity to AITC or capsaicin

A weak correlation was found between AITC and capsaicin cough sensitivity in the log C2 (r = 0.200, *p* = 0.002) and log C5 (r = 0.349, *p* = 0.001) values of patients with chronic refractory cough. There was also a weak correlation between AITC and capsaicin in the log C2 values of healthy subjects (r = 0.399, *p* = 0.003) but not for the log C5 values (r = 0.029, *p* = 0.835).

### Comparison of patients with chronic refractory cough and healthy subjects in AITC and capsaicin cough sensitivity

**Fig. 3:**
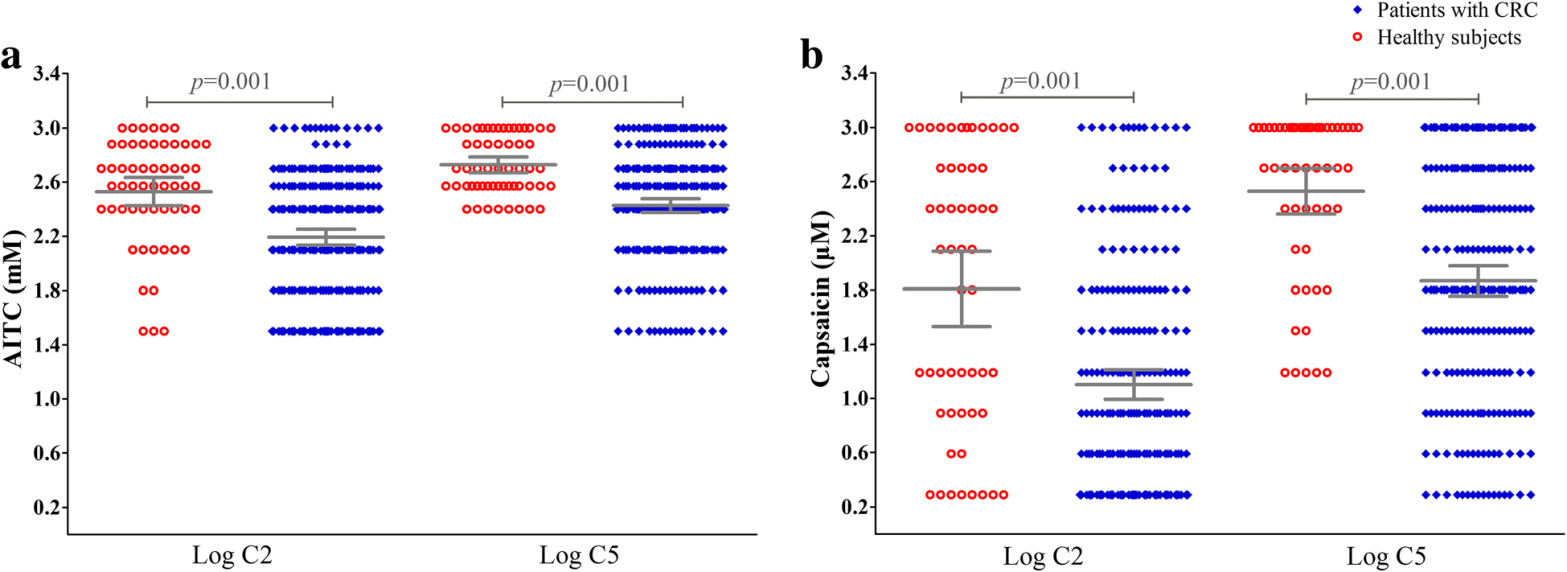
Individual AITC and capsaicin cough sensitivity measured as C2 or C5. AITC (Panels **a**), capsaicin (Panels **b**). C2: the lowest concentrations of AITC or capsaicin which evoked two to four coughs; C5: the lowest concentrations of AITC or capsaicin which evoked five coughs or more. Horizontal lines and error bars represent mean values and 95% confidence intervals of log C2 or log C5

Patients with chronic refractory cough had an increased cough sensitivity (mean [95% confidence interval]) to AITC compared to that of the healthy subjects (log C2: 2.19 [2.13–2.25] vs 2.53 [2.42–2.64] mM, *p* = 0.001; log C5: 2.42 [2.37–2.48] vs 2.72 [2.66–2.78] mM, *p* = 0.001). For the capsaicin challenge, patients with chronic refractory cough had an increased cough sensitivity compared to that of the healthy subjects (log C2: 1.10 [0.99–1.21] vs 1.84 [1.56–2.11] μM, *p* = 0.001; log C5: 1.87 [1.75–1.98] vs 2.53 [2.36–2.70] μM, *p* = 0.001) (Fig. 3).

### Comparison of female and male subjects’ AITC and capsaicin cough sensitivities

**Fig. 4:**
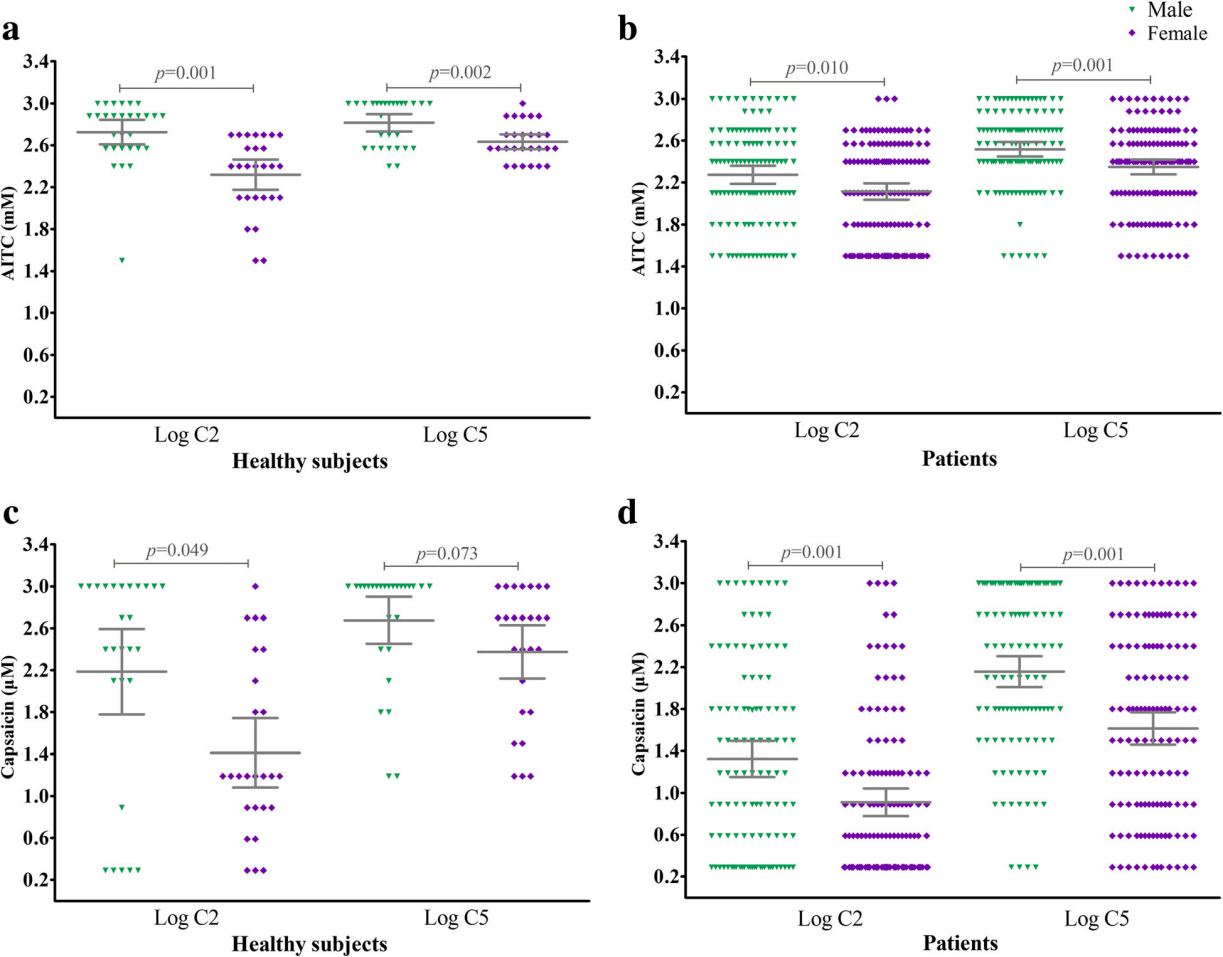
Gender difference in AITC and capsaicin cough sensitivity measured as C2 or C5. AITC (Panels **a** and **b**), capsaicin (Panels **c** and **d**). C2: the lowest concentrations of AITC or capsaicin which evoked two to four coughs; C5: the lowest concentrations of AITC or capsaicin which evoked five coughs or more. Horizontal lines and error bars represent mean values and 95% confidence intervals of log C2 or log C5

Female subjects manifested a heightened AITC cough sensitivity compared to that of male subjects both in patients with chronic refractory cough (log C2: 2.11 [2.03–2.19] vs 2.27 [2.19–2.36] mM, *p* = 0.010; log C5: 2.35 [2.28–2.42] vs 2.52 [2.45–2.59] mM, *p* = 0.001) and healthy subjects (log C2: 2.32 [2.18–2.46] vs 2.72 [2.61–2.84] mM, *p* = 0.001; log C5: 2.63 [2.56–2.71] vs 2.82 [2.73–2.90] mM, *p* = 0.002). In addition, female subjects showed an increased capsaicin cough sensitivity compared to that of male subjects (Patients: log C2: 0.91 [0.78–1.04] vs 1.32 [1.15–1.49] μM, *p* = 0.001; log C5: 1.61 [1.46–1.77] vs 2.16 [2.01–2.30] μM, *p* = 0.001. Healthy subjects: log C2 1.41 [1.08–1.74] vs 2.19 [1.78–2.60] μM, *p* = 0.049; log C5: 2.38 [2.12–2.63] vs 2.68 [2.45–2.90] μM, *p* = 0.073) (Fig. 4).

### Safety

In preliminary experiments in healthy subjects or patients with chronic refractory cough, there was no difference in pulmonary function before and after the AITC cough challenge (all *p* > 0.05) (Table 4). Adverse effects were reported by 16 (6.40%) of the 250 patients (throat irritation: *n* = 9; mild nausea: *n* = 4; mild chest tightness: *n* = 3) and 3 of the 56 (5.4%) healthy subjects (throat irritation: *n* = 2; mild nausea: *n* = 1) during the AITC cough challenge, as well as 13 (5.6%) of the 234 patients (throat irritation: *n* = 7; mild nausea: *n* = 3; mild chest tightness: *n* = 3) and 3 of the 53 (5.7%) healthy subjects (throat irritation: *n* = 1; mild nausea: *n* = 2) during the capsaicin challenge. All complaints spontaneously resolved within 30 min.

**Table 4:** Pulmonary function at baseline and post AITC cough challenge of subjects

	Healthy subjects			Patients with CRC		
	Baseline	Post challenge	*P*	Baseline	Post challenge	*P*
FEV_1_ (L)	3.5 ± 0.4	3.5 ± 0.4	0.733	3.6 ± 0.8	3.5 ± 0.8	0.168
FEV_1_% pred	102.4 ± 12.2	102.3 ± 12.7	0.808	96.3 ± 7.8	95.6 ± 8.1	0.133
FVC (L)	4.0 ± 0.6	4.0 ± 0.6	0.421	4.2 ± 0.9	4.2 ± 0.9	0.226
FVC % pred	100.4 ± 13.1	100.2 ± 13.0	0.590	95.8 ± 9.1	96.9 ± 10.2	0.129
FEV_1_ / FVC	86.5 ± 4.9	86.5 ± 5.0	0.991	85.4 ± 5.8	85.3 ± 6.4	0.879
MMEF (L)	4.2 ± 0.7	4.2 ± 0.9	0.116	3.9 ± 1.5	3.9 ± 1.4	0.746
MMEF % pred	100.5 ± 20.1	100.3 ± 20.2	0.205	83.6 ± 19.8	83.5 ± 17.1	0.915

Data are expressed as mean ± SD. *CRC* chronic refractory cough, *FEV*_*1*_ forced expiratory volume in 1 s, *FVC* forced vital capacity, *MMEF* maximum mid-expiratory flow

## Discussion

This is the first study to investigate the phenotypes of cough hypersensitivity by examining TRPA1- and TRPV1-mediated cough sensitivity in patients with chronic refractory cough. AITC is a common, naturally occurring isothiocyanate [18], which has been reported to possess antioxidant, antimicrobial, and anticancer properties [19, 20]. The consumption of 6.0 mg AITC did not present a problem for several age groups in a population [19]. In our study, the maximal concentration of AITC solution (1000 mM) was equivalent to a very small dose of 0.0025 mg of AITC. In addition, AITC nasal challenge has been performed in patients with idiopathic rhinitis and healthy volunteers without causing any serious side effects [21, 22]. We also found that AITC was well tolerated by participants and did not cause bronchoconstriction. Thus, AITC cough challenge is a safe method to assess cough reflex sensitivity.

We identified a distinct concentration–response relationship to inhaled AITC. C5 was reached in 95.6% of patients with chronic refractory cough and 92.9% of healthy subjects using up to the highest concentration (1000 mM) of AITC solution. In addition, patients with chronic refractory cough manifested a heightened cough response to AITC compared to that of healthy subjects. Therefore, AITC cough challenge is able to detect TRPA1-mediated cough sensitivity in patients with chronic refractory cough. Furthermore, TRPA1, but not TRPV1, can be activated by environmental irritants, such as ozone, isothiocyanate, cinnamaldehyde, acrolein and nicotine, many of which are present in air pollution, vehicle exhaust and cigarette smoke [23, 24]. Considering the fact that studies have confirmed a link between occupational and environmental exposures and persistent cough [25, 26], along with the important role of TRPA1 in workplace and air pollution-related inflammatory diseases, the introduction of AITC cough challenge would supplement the use of capsaicin cough challenge.

The weak correlation between AITC and capsaicin cough sensitivity in patients might be attributed to co-expressed TRPV1 and TRPA1 in vagal pulmonary C-fibre sensory nerves [27]. It is highly probable that TRPV1 and TRPA1 will be activated simultaneously during episodes of airway inflammation. Moreover, a distinct potentiating effect through the simultaneous activation of TRPA1 and TRPV1 by their respective selective agonists was found in rats [28].

A number of studies have shown that patients with chronic cough are, on average, more sensitive to inhaled tussigenic agents than healthy controls, but there has been a significant overlap in the C5 values of these tussigenic agents between groups [29, 30]. To define hypertussivity to each of the challenges, we defined increased cough sensitivity as a log C5 value lower than that of all control subjects. Thus, we found an increased cough sensitivity to AITC in 29.5% of patients with chronic refractory cough, while 22.6% of patients exhibited increased cough sensitivity to capsaicin. Combining the two challenge tests, cough hypersensitivity to these agents was found in 41.4% of patients. In animal studies, TRPA1 antagonists and TRPV1 antagonists have been shown to have a high degree of antitussive efficacy [31, 32]. However, neither TRPA1 nor TRPV1 antagonists have been shown to reduce cough in patients with chronic refractory cough in recent clinical studies [33, 34], questioning the hypothesis that blockade of the TRPA1 or TRPV1 receptor might ameliorate chronic refractory cough. Our results may provide another reason for this lack of efficacy. In light of the results showing that as few as 18.8% of patients with chronic refractory cough showed hypersensitivity to AITC only, 11.9% of patients showed hypersensitivity to capsaicin only, and 58.6% of patients did not manifest hypersensitivity to AITC or capsaicin, a minority of patients might have benefited from these channel blockers. Animal studies are therefore not reliable to predict therapy responses in clinical practice since an improvement in cough reflex sensitivity to a specific agonist after treatment with a single antagonist is often the primary endpoint, which could hardly mimic cough hypersensitivity in humans because of the diverse mechanisms of activation.

We found no differences in the characteristics of the cough patients with TRPA1- and TRPV1-mediated cough hypersensitivity and those without this hypersensitivity with regard to their cough triggers and the presence of laryngeal paraesthesia. This finding indicates that uncontrollable bouts of coughing triggered by low-level physical or chemical stimuli were a general clinical feature of patients with chronic refractory cough. However, the most interesting difference was that while patients without heightened cough sensitivity to capsaicin or AITC were predominantly male, those with hypersensitivity were predominantly female. A higher cough sensitivity in females compared to males has been reported in previous studies using capsaicin, citric acid or tartaric acid as tussive agents [35–38]. We can now add that females with chronic cough are also more likely to have cough hypersensitivity to TRPA1 agonists. The mechanism underlying the female predominance of AITC hypersensitivity deserves further elucidation, but one study has indicated that this hypersensitivity might be related to a larger somatosensory response to inhaled capsaicin in females [39].

The heterogeneity of cough hypersensitivity responses has been reported previously, highlighting differences between chronic refractory cough and other airway conditions such as asthma and chronic obstructive pulmonary disease (COPD). Thus, patients with COPD exhibited an enhanced cough response to capsaicin with a diminished cough response to PGE_2_, while patients with asthma demonstrated an enhanced response to citric acid, and patients with chronic cough showed a heightened response to both capsaicin and citric acid [30]. Our study indicates that there is also heterogeneity of cough hypersensitivity to TRP receptors, even within chronic refractory cough patients. This heterogeneity of cough hypersensitivity might form the basis for various phenotypes of chronic cough [5].

There is increasing evidence indicating that the pathways underlying the development and persistence of chronic cough are heterogeneous with multiple neural pathways capable of eliciting cough [5, 40]. The adenosine triphosphate (ATP)-gated P2X3-containing receptors have recently gained attention as potential therapeutic targets for the treatment of chronic refractory cough [41, 42]. P2X3 forms homotrimeric P2X3 receptors (consisting of three P2X3 subunits) or P2X2/P2X3 receptors (consisting of two P2X3 subunits and one P2X2 subunit) [43]. Selective antagonists of homotrimeric P2X3 receptors seem more promising given that growing evidence suggests that homotrimeric P2X3 receptors are responsible for the hypersensitization of the cough reflex, while inhibition of P2X2/P2X3 receptors are responsible for taste-related side-effects [44]. There is also evidence supporting a role of TRPV4 in physiological and pathophysiological processes of cough [45]. The TRPV4-ATP-P2X3 interaction is a possible contributor to the abnormally upregulated cough reflex since it has been reported that TRPV4 can cause the release of ATP, which in turn acts on sensory nerves expressing P2X3 [46]. In addition, a P2X3 receptor antagonist, gefapixant, blocks both P2X3-mediated and TRPV4-mediated cough in patients with chronic refractory cough [47]. We speculate that those patients who did not present hypersensitivity to TRPV1 or TRPA1 agonists might be sensitive to other receptor agonists or antagonists. Further investigations are needed to define the pathways of cough hypersensitivity, particularly in those who are not hypersensitive to both TRPA1 and TRPV1 agonists.

Our study has some limitations. Because AITC is relatively insoluble in water, we used ethanol to dissolve it, and ethanol might augment the cough response in sensitive individuals [48, 49]. To rule out the potential effect of ethanol, each subject inhaled a control solution of 50% ethanol prior to the AITC solution. We found few patients coughed after inhaling a 50% ethanol solution. In addition, a concentration–dependent induction of cough by AITC was demonstrated. We concluded that ethanol did not interfere with the AITC challenge, although we cannot completely exclude its effects.

## Conclusions

In conclusion, we have established an AITC cough challenge to test TRPA1-mediated cough sensitivity in patients with chronic refractory cough and in healthy subjects. This challenge strongly supplements the widely used capsaicin cough challenge. Cough hypersensitivity to TRPV1 or TRPA1 agonists alone or jointly can be demonstrated, and TRPA1- and TRPV1-mediated cough hypersensitivity was absent in half of the patients. These results reflect the heterogeneity of cough hypersensitivity and indicate a direction for personalized management of patients with chronic refractory cough.

## Data Availability

The datasets used and/or analysed during the current study are available from the corresponding author on reasonable request.

## Abbreviations

AITC: Allyl isothiocyanate
C2: the lowest concentrations of AITC or capsaicin which evoked two to four coughs
C5: the lowest concentrations of AITC or capsaicin which evoked five coughs or more
TRPA1: Transient receptor potential ankyrin 1
TRPV1: Transient receptor potential vanilloid 1
